# Synthesis, Structural Property, Photophysical Property, Photocatalytic Property of Novel ZnBiErO_4_ under Visible Light Irradiation

**DOI:** 10.3390/ma11020303

**Published:** 2018-02-18

**Authors:** Jingfei Luan, Yan Zhuang

**Affiliations:** 1 School of Chemistry and Environmental Engineering, Changchun University of Science and Technology, Changchun 130600, China; 2 State Key Laboratory of Pollution Control and Resource Reuse, School of the Environment, Nanjing University, Nanjing 210093, China; yanzhuang1011@yahoo.com

**Keywords:** ZnBiErO_4_, photocatalyst, methyl blue, photocatalytic degradation, visible light irradiation

## Abstract

A novel photocatalyst ZnBiErO_4_ was firstly synthesized by solid-state reaction method and its structural and photocatalytic properties were analyzed by scanning electron microscopy, X-ray diffraction, X-ray photoelectron spectroscopy and UV-Vis diffuse reflectance. The results demonstrated that ZnBiErO_4_ crystallized with tetragonal crystal structure with space group I41/A. The lattice parameters for ZnBiErO_4_ were proved to be a = b = 10.255738 Å and c = 9.938888 Å. The band gap of ZnBiErO_4_ was estimated to be about 1.69 eV. Compared with nitrogen doped TiO_2_, ZnBiErO_4_ showed excellent photocatalytic activities for degrading methyl blue during visible light irradiation. The photocatalytic degradation of methyl blue with ZnBiErO_4_ or N-doped TiO_2_ as catalyst followed the first-order reaction kinetics. Moreover, the apparent first-order rate constant of ZnBiErO_4_ or N-doped TiO_2_ was 0.01607 min^−1^ or 0.00435 min^−1^. The reduction of total organic carbon, formation of inorganic products, such as SO_4_^2−^ and NO_3_^−^ and the evolution of CO_2_ revealed the continuous mineralization of methyl blue during the photocatalytic process. ZnBiErO_4_ photocatalyst had great potential to purify textile industry wastewater.

## 1. Introduction

The organic pollutants of dye wastewater formatted by the industrial production, are toxicity, unacceptable color, high chemical oxygen demand and biological degradation, which caused the difficulty of self-degradation in natural conditions [1]. It will not only get undesirable result but also produce benzene, aniline and other secondary pollutants sometimes when we degrade it by traditional treatment methods [2,3,4,5,6,7]. In recent years, photocatalysis has caused general concern because of its strong oxidizability to degrade many organic pollutants in wastewater and effluents, especially for the dyes wastewater [8,9,10,11,12,13,14,15]. In recent years, as a semiconductor, TiO_2_ photocatalyst has attracted wide attention owing to the property of non-toxic, excellent stability and low cost [16,17,18]. However, due to the wide band gap (3.2 eV) of TiO_2_, the required wavelength is less than 387.5 nm, which near ultraviolet light and occupies only 4~5% of the solar energy and thus limits its application [19,20]. Although a variety of methods have been tried to modify TiO_2_, its quantum efficiency is still very low and the modification brings more shortcomings [21,22,23,24,25,26]. Based on the reason, it is necessary to develop new visible light responsive photocatalysts to utilize more visible light, which accounts for about 43% of the solar energy. Therefore, it is of grate important to develop the catalysts which can response to visible light. And many scientists have engaged in photocatalysis using visible light today [27,28,29,30,31,32,33,34,35].

AB_2_O_4_-type spinel composite oxides with narrow band gap have attracted extensive attention because of its excellent visible light response in textile wastewater treatment in recent years. They are capable to be used in adsorption, photocatalysis, hydrogen production and chemical sensors. It is demonstrated that NiCo_2_O_4_ and ZnFe_2_O_4_ exhibited a good photocatalytic activity for degrading methylene blue and other organic under visible light irradiation [36,37]. CoAl_2_O_4_ films were prepared in different structure, composition, morphology and optical properties by the method of chemical vapor deposition in 800–1200 °C by Carta et al. and its high photocatalytic activity were demonstrated [38]. The spinel oxide CaBi_2_O_4_ synthesized by Tang et al. could not only degrade gas phase acetaldehyde and MB aqueous solution under visible light irradiation but also keep high catalyst recovery [39]. ZnCo_2_O_4_ nanoparticles were synthesized by a co-precipitation decomposition method and the optical band gap of the nanoparticles was estimated to be 3.39 eV from the UV-Vis absorption spectrum. On the basis of the laboratory test results, the ZnCo_2_O_4_ nanoparticles performed a high photocatalytic activity for the degradation of methyl blue dye solution under visible light irradiation [40]. ZnBiGaO_4_ was prepared to photocatalytic decompose hydrogen sulfide (H_2_S) with two different methods under visible light irradiation, its band gap was estimated to be 2.8 eV [41]. MgFe_2_O_4_/PANI photocatalyst, which was synthesized by hydrothermal synthesis method, proved a high effective photodecolorization of both methylene blue and methyl orange dyes in aqueous solution and maintained a high removal ability after five repeated utilizations [42].

ZnBiErO_4_ is a material that has never been synthesized and the data about its structural and photocatalytic properties such as space group and lattice constants have not been found before. ZnBiErO_4_ seems to have a potential to improve the photocatalytic activity, because it has been proved that a slight modification of a semiconductor structure will result in a remarkable change in photocatalytic properties [43]. Herein, ZnBiErO_4_ was prepared by the solid-state reaction method for the first time. We studied its structure and photocatalytic properties through a series of characterizations. Photocatalytic performances of ZnBiErO_4_ in the degradation of methyl blue (MB) under visible light were also investigated. At the same time, we made a comparison between ZnBiErO_4_ and N-doped titanium dioxide to discover their differences in photocatalytic activity. The experimental results showed that the catalytic system with ZnBiErO_4_ revealed unusual catalytic effects for MB under visible light irradiation. 

## 2. Results and Discussion

### 2.1. Characterization

**Figure 1:**
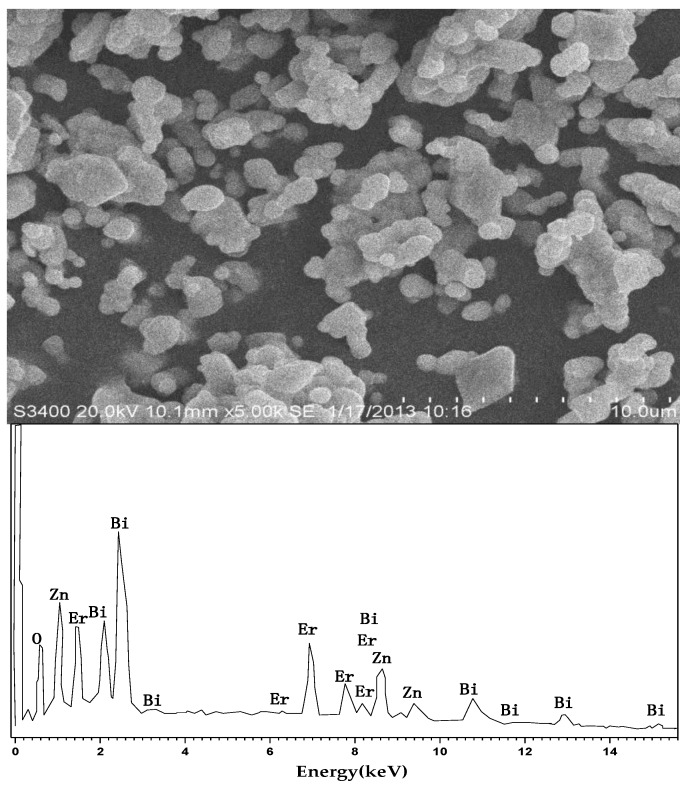
SEM-EDS spectrum of ZnBiErO_4_.

**Figure 2:**
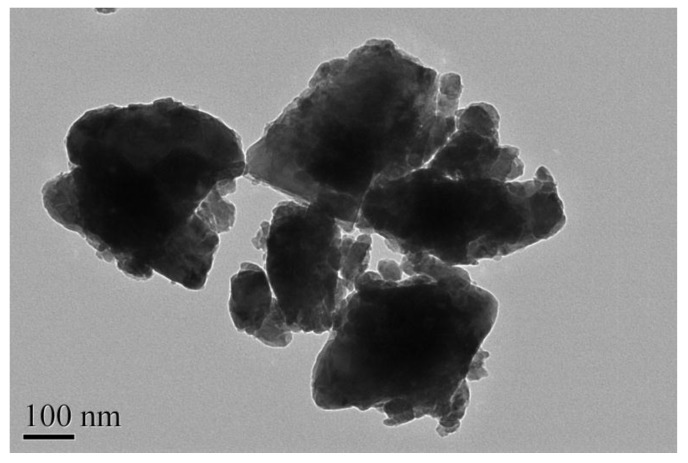
TEM image of ZnBiErO_4_.

**Figure 3:**
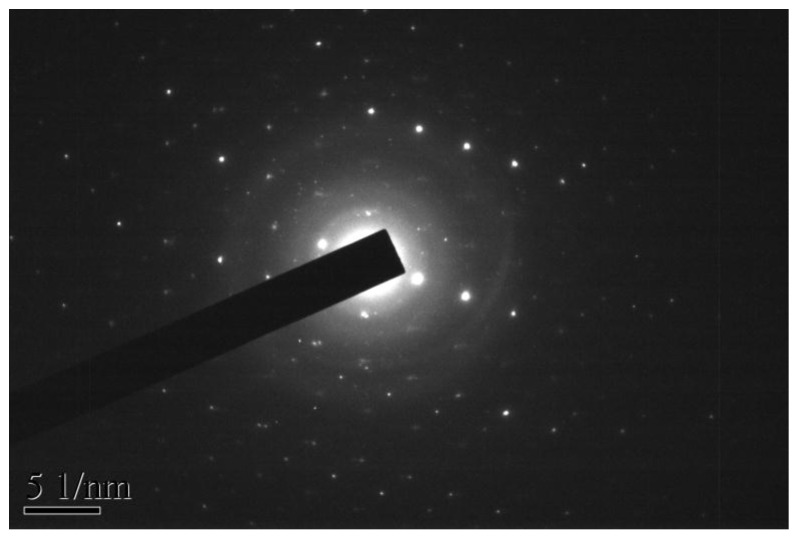
The selected area electron diffraction pattern of ZnBiErO_4_.

**Figure 4:**
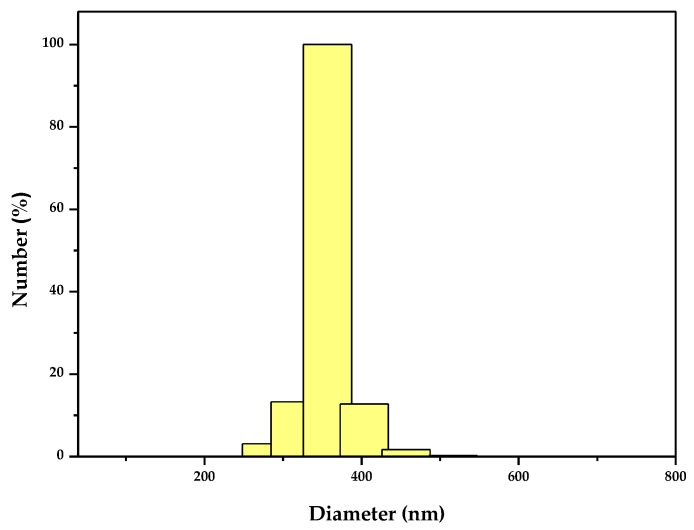
The diagram of particle size of ZnBiErO_4_.

Figure 1 is the scanning electron microscope-X-ray energy dispersion (SEM-EDS) spectrum of the as-synthesized ZnBiErO_4_. The composition was analyzed by EDS and the result was in line with that of XRD patterns. According to the SEM-EDS spectrum, the prepared ZnBiErO_4_ was composed of the elements of zinc, bismuth, Erbium and oxygen, furthermore, the mean atomic ratio of Zn:Bi:Er:O was estimated to be 1:1:1:4. Fortunately, other elements were not traced, which showed that our prepared ZnBiErO_4_ catalyst was pure phase without any other impurities. The SEM image shows the morphology of ZnBiErO_4_ particle and the irregular shape and uniform particle size of ZnBiErO_4_ were observed. Figure 2 shows a typical transmission electron microscopy (TEM) image of ZnBiErO_4_ catalyst, which reveals that the average particle diameter of ZnBiErO_4_ was about 350 nm. The corresponding selected-area electron diffraction (SAED) pattern is presented in Figure 3, showing discrete diffraction rings. It is suggested that the single sheet was comprised by multi crystals. Figure 4 is the diagram of particle size of ZnBiErO_4_ catalyst, which indicated that the particle size of ZnBiErO_4_ was mainly distributed in 326~387 nm. The result was in line with the TEM.

**Figure 5:**
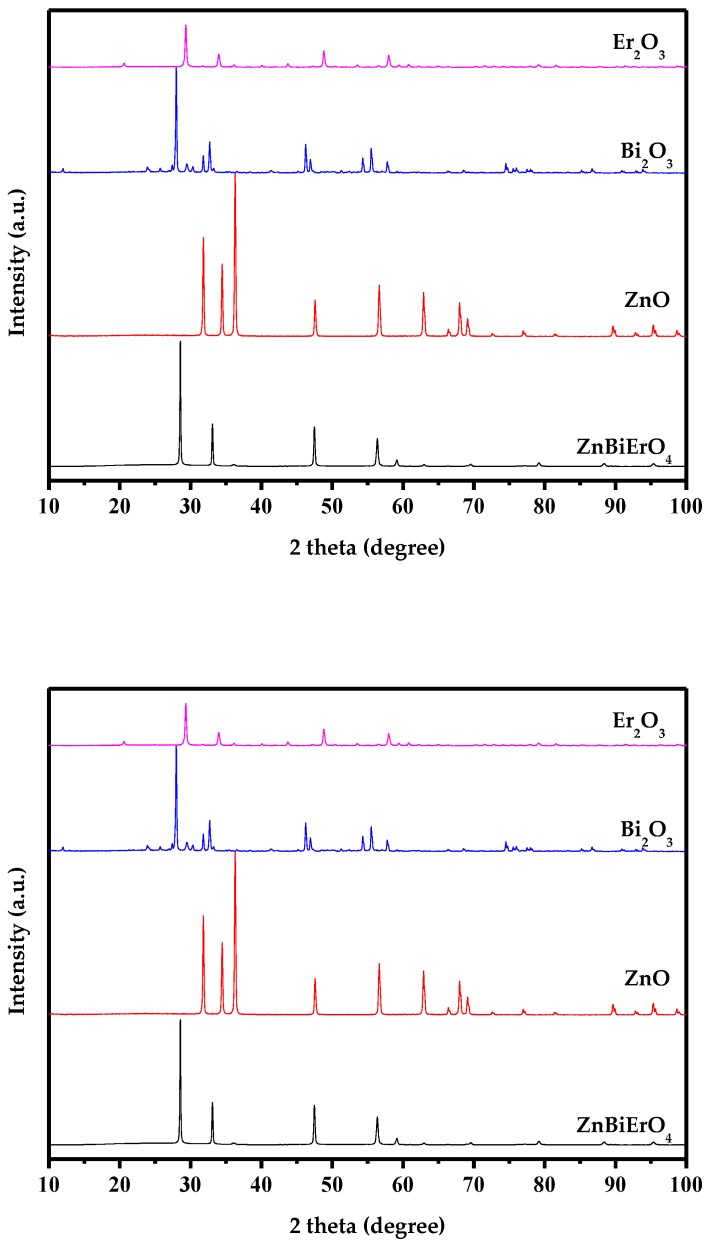
X-ray powder diffraction patterns of ZnBiErO_4_, ZnO, Bi_2_O_3_ and Er_2_O_3_.

**Figure 6:**
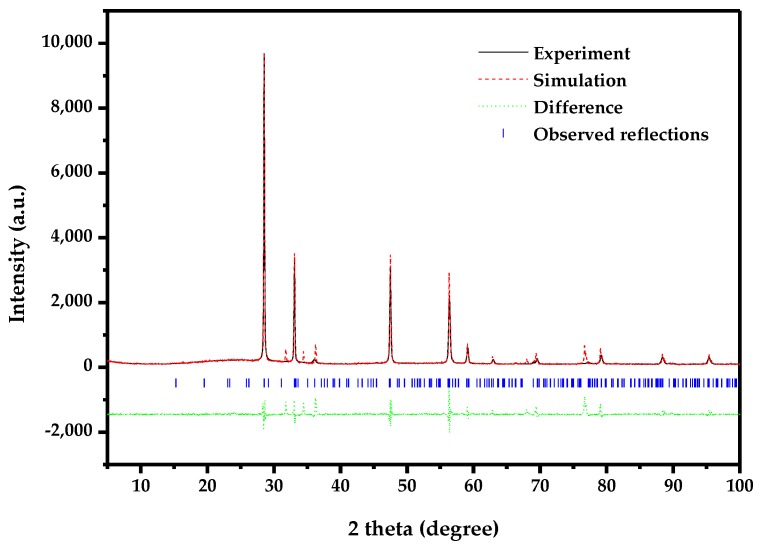
Pawley refinements of XRD data for novel photocatalyst ZnBiErO_4_ prepared by the solid-state reaction method at 1000 °C. The solid line represents experimental X-ray diffraction pattern. The dot line represents simulation X-ray diffraction pattern. The tic marks represent reflection position. A difference between observed and calculated profile is shown beneath.

**Figure 7:**
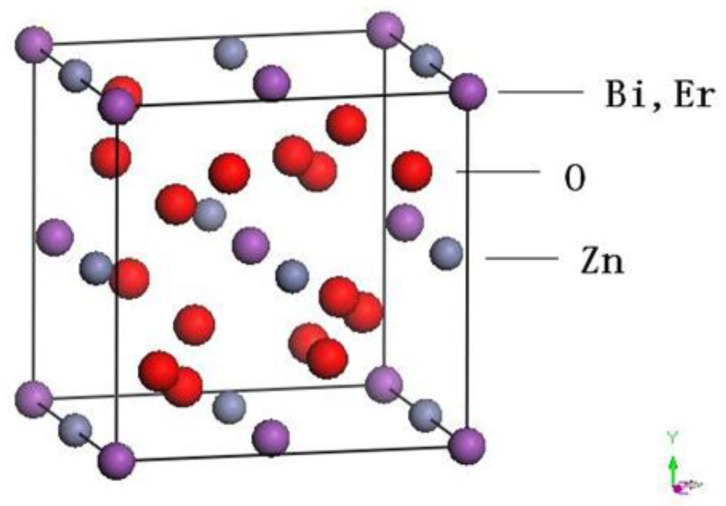
Atomic structure of ZnBiErO_4_.

Figure 5 represents the X-ray powder diffraction patterns of ZnBiErO_4_, ZnO, Bi_2_O_3_ and Er_2_O_3_. Compared to the individual oxides used in synthesis, the synthesized photocatalyst was proved to be novel and it was not the mixture of individual oxides sintered at high temperature. Full-profile structure refinements of the collected X-ray diffraction data of ZnBiErO_4_ were continued to investigate by the RIETAN^TM^ program, which was based on Pawley analysis. The obtained XRD patterns of ZnBiErO_4_ samples are shown in Figure 6. It is demonstrated that the observed intensities were in according with the calculated intensities in a tetragonal crystal structure with space group I41/A. The lattice parameters for ZnBiErO_4_ were proved to be a = b = 10.255738 Å and c = 9.938888 Å. According to the lattice constants and the space group I41/A. All of the diffraction peaks for ZnBiErO_4_ could be successfully indexed and no other impurity phases are found, showing the single phase of the products. The result was in line with SEM-EDS spectrum. In order to simulate the structure of ZnBiErO_4_ accurately, Pawley refinements of the XRD spectrum and the three-dimensional space structure model of ZnBiErO_4_ photocatalyst were established by Material Studio software (Material Studio 2.0, Accelrys Corporation, USA). The atomic coordinates and structural parameters of ZnBiErO_4_ are listed in Table 1 and the atomic structure of ZnBiErO_4_ is shown in Figure 7. 

**Figure 8:**
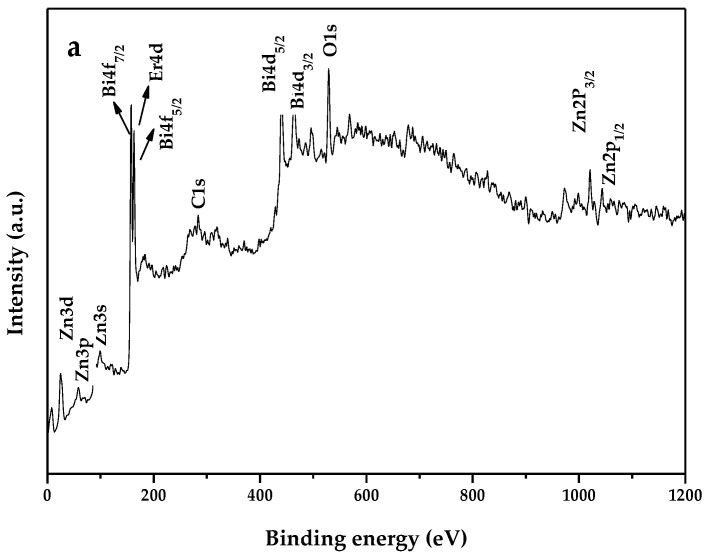

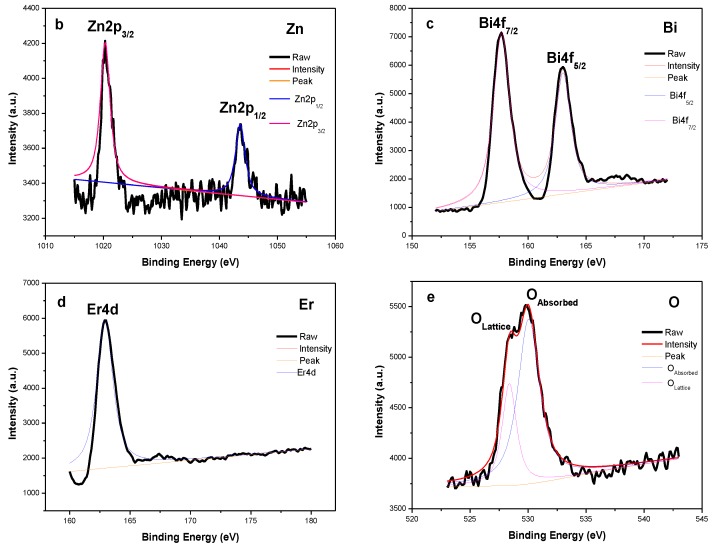
(**a**) The XPS full spectrum of ZnBiErO_4_; (**b**) Zn2p spectrum of ZnBiErO_4_; (**c**) Bi4f spectrum of ZnBiErO_4_; (**d**) Er4d spectrum of ZnBiErO_4_; (**e**) O1s spectrum of ZnBiErO_4_.

In order to get the surface chemical compositions and the valence states of various elements of ZnBiErO_4_, the X-ray photoelectron spectrum (XPS) was carried out. The XPS full spectrum and detailed spectrum of ZnBiErO_4_ are shown in Figure 8. To see the results clearly, various elemental peaks with specific binding energies are provided in Table 2. According to the XPS full spectrum in Figure 8a, the synthesized ZnBiErO_4_ contained the elements of Zn, Bi, Er, O and C. The element of C was come from hydrocarbon for the sake of testing, so it was in agreement with the results of SEM-EDS. By fitting and separating the peak, we got the XPS detailed spectrum in Figure 8b–e. Zn2p_1/2_ and Zn2p_3/2_ peaks of Zn element were seen in Figure 8b, whose positions were 1043.6 eV and 1023.3 eV, respectively. Bi4f_7/2_ and Bi4f_5/2_ peaks of Bi element were observed in Figure 8c, whose positions were 157.7 eV and 163.0 eV, respectively. And the Er4d peak of Er element was presented in Figure 8d, whose position was 162.9 eV. It was obvious to find that there existed shoulders peaks at the XPS spectrum of O1s. By fitting and separating the peak, we got O_Lattice_ peak and O_Absorbed_ peak, which indicated that the catalyst absorbed some O_2_ in the process of calcination. Otherwise, the results showed that the oxidation states of Zn, Bi, Er and O ions from ZnBiErO_4_ were +2, +3, +3 and −2, respectively. Therefore, it could be deduced that the resulting material was highly pure under our preparation conditions. Notably, neither shoulders nor widening in the XPS peaks of ZnBiErO_4_ was observed, indicating the absence of any other phases.

**Figure 9:**
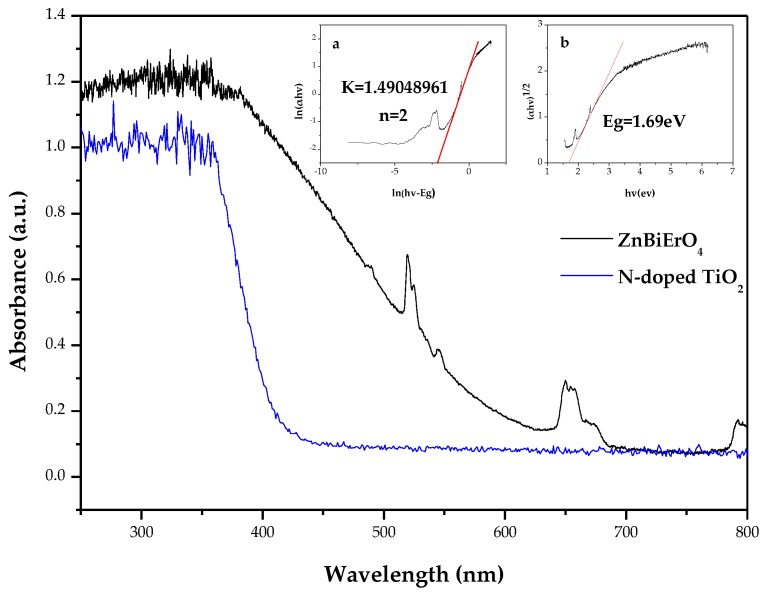
UV-Vis absorption spectra of ZnBiErO_4_ and N-doped TiO_2_. (**a**) plot of ln(*αhν*) versus ln(*hν − E*_g_) for ZnBiErO_4_. (**b**) plot of (*αhν*)^1/2^ versus *hν* for ZnBiErO_4_.

The UV-vis absorption spectra of ZnBiErO_4_ and N-doped TiO_2_ are shown in Figure 9. In contrast to the N-doped TiO_2_, whose absorption edge was 414 nm, the novel synthesized ZnBiErO_4_ photocatalyst exhibited an obvious absorption in the visible region up to 690 nm. Furthermore, the band gap of ZnBiErO_4_ and N-doped TiO_2_ by extrapolating the linear portion of the curve to zero absorbance are 1.69 eV and 2.76 eV, respectively. It indicated that ZnBiErO_4_ could be excited by visible light and might exhibit a better visible light photocatalytic activity. Apparently, reflection and scattering did not cause a significant absorption.

For a crystalline semiconductor, the optical absorption near the band edge followed the equation [44]:

*αhν* = A(*hν* − *E_g_*)*^n^* (1)

Here, A, *α*, *E*_g_ and *ν* are proportional constant, absorption coefficient, band gap and light frequency, respectively. Within this equation, *n* determines the character of the transition in a semiconductor. *E*_g_ and *n* can be calculated by the following steps: (i) plotting ln(*αhν*) versus ln(*hν* − *E*_g_) by assuming an approximate value of *E*_g_ (ii) deducing the value of *n* based on the slope in this graph. (iii) refining the value of *E*_g_ by plotting (*αhν*)^1*/n*^ versus *hν* and extrapolating the plot to (*αhν*)^1*/n*^ = 0. Figure 9a shows the plot of ln(*αhν*) versus ln(*hν − E*_g_) for ZnBiErO_4_. Based on the Figure 9a, the slope of the graph was 1.49048961 and the value of *n* for ZnBiErO_4_ was calculated to be 2. Figure 9b shows the plot of (*αhν*)^1*/*2^ versus *hν* for ZnBiErO_4_. It was obvious to see that the value of *E*_g_ for ZnBiErO_4_ was calculated to be 1.69 eV, indicating that ZnBiErO_4_ possessed narrow band gap and the optical transition for ZnBiErO_4_ was directly allowed.

### 2.2. Photocatalytic Activity 

**Figure 10:**
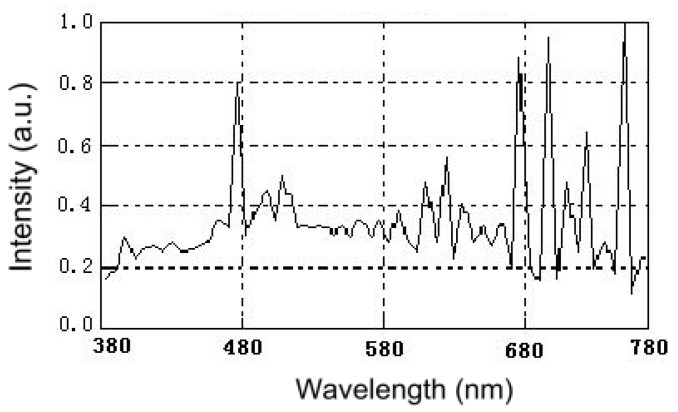
The irradiance spectrum of the Xeon lamp light (cited from [45]).

**Figure 11:**
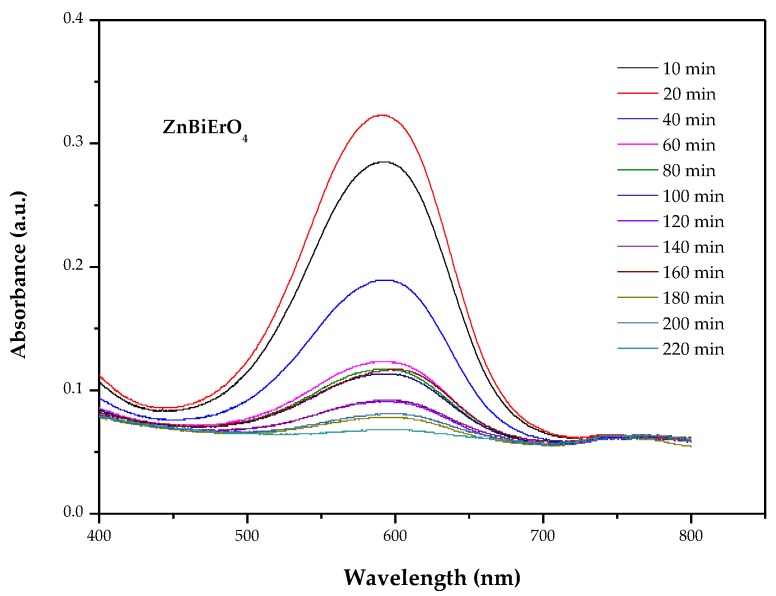
Temporal spectral changes of aqueous solutions of methyl blue due to light irradiation in the presence of ZnBiErO_4_.

Figure 10 (cited from [45]) shows the irradiance spectrum of the xenon lamp light. From the diagram, we can learn that the spectrum of xenon lamp is close to the visible light. Therefore, a 500 W xenon lamp was used as a light source with a 400 nm cutoff filter to provide visible light irradiation in this paper. Figure 11 shows temporal spectral changes of aqueous solutions of methyl blue (MB) due to visible light irradiation in the presence of ZnBiErO_4_. The result illustrated that ZnBiErO_4_ could photodegrade MB effectively under visible light irradiation and the color of MB solution shallowed gradually when irradiation time increased. Otherwise, it was also obvious to observe that there was a significant reduction in typical MB peaks in 591 nm from the results, demonstrating that the maximum absorption wavelength of MB was 591 nm. The result is close to the study by Cai Jiabia et al., in which the absorbance wavelength of methyl blue is 596 nm [46].

**Figure 12:**
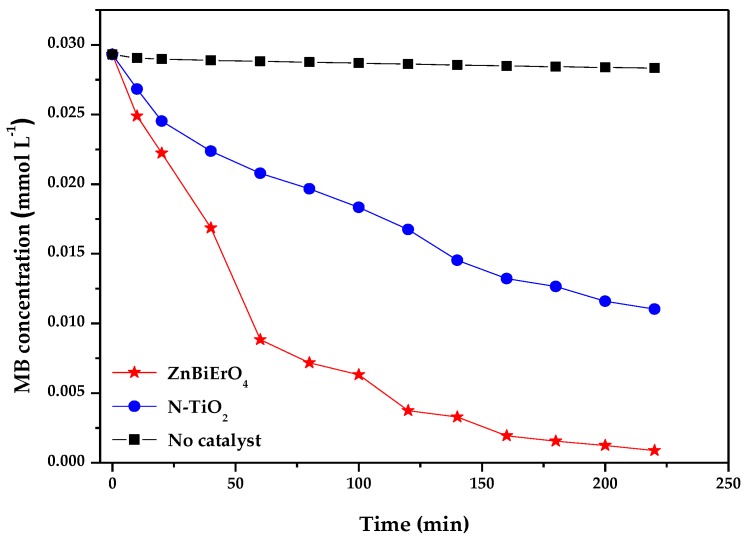
Photocatalytic degradation of methyl blue under visible light irradiation in the presence of ZnBiErO_4_ and N-TiO_2_ as well as in the absence of a photocatalyst.

According to the principle of photocatalysis, electron-hole pairs within ZnBiErO_4_ resulted from the direct absorption of band-gap photons began to diffuse to the surface of ZnBiErO_4_, as a result, the photocatalytic activity of ZnBiErO_4_ for degradation of organic compounds might be enhanced. Figure 12 describes the photocatalytic degradation chart of MB under visible light irradiation in the presence of ZnBiErO_4_, N-doped TiO_2_ (N-TiO_2_) as well as in the absence of a photocatalyst. Based on the changes in the UV-Vis spectrum of decomposing MB in visible light (*λ* > 400 nm) by ZnBiErO_4_, N-doped TiO_2_, it could be seen that both ZnBiErO_4_ and N-doped TiO_2_ could photodegrade MB effectively under visible light irradiation. After visible light irradiation for 220 min, the removal rate of MB was estimated to be 96.99%, 62.39% or 3.31% with ZnBiErO_4_, N-doped TiO_2_ as catalyst, as well as in the absence of a photocatalyst, respectively. From the Figure 12, we can observe that the speed was slower of MB degradation by using ZnBiErO_4_ or N-doped TiO_2_ as a photocatalyst during the later reaction process compared with the speed of 0–60 min. According the results, we supposed that some intermediate products generated during MB degradation occupied active reaction sites on the photocatalytic surface, which could reduce the number of oxygen active radical and the reaction probability between the dye of MB and optical electron-hole pairs [47,48,49]. The photodegradation rate of MB was about 2.153 × 10^−9^ mol L^−1^ s^−1^ or 1.385 × 10^−9^ mol L^−1^ s^−1^ with ZnBiErO_4_ or N-doped TiO_2_ as catalyst under visible light irradiation for 220 min. The self-degradation rate of MB was 0.07348 × 10^−9^ mol L^−1^ s^−1^ without a catalyst. Furthermore, the photonic efficiency and the quantum yield was estimated to be 0.0452% (*λ* = 420 nm) and 0.6134% by using ZnBiErO_4_ as catalyst. Similarly, the photonic efficiency and the quantum yield was estimated to be 0.0291% (*λ* = 420 nm) and 0.3946% with N-doped TiO_2_ as catalyst. It could obviously tell that the photodegradation rate of MB, the photonic efficiency and quantum yield with ZnBiErO_4_ as catalyst were higher than those with N-doped TiO_2_ as catalyst, indicating that the photocatalytic degradation activity of ZnBiErO_4_ under visible light was higher than N-doped TiO_2_. One of the reason might be that a high photonic efficiency and quantum yield could lead to the production of a large number of electron-hole pairs, which were responsible for the photocatalytic degradation reaction directly or indirectly [50]. In addition, the band gap was another factor [51]. The band gap of ZnBiErO_4_ was calculated to be 1.69 eV and the band gap of N-doped TiO_2_ was 2.76 eV, demonstrated that ZnBiErO_4_ possessed narrower band gap compared with N-doped TiO_2_. Therefore, ZnBiErO_4_ could make use of more irradiation light energy than N-doped TiO_2_, which was more beneficial to generate separate photogenerated electrons and photogenerated holes. Subsequently, the ZnBiErO_4_ photocatalytic activities were improved. In conclusion, ZnBiErO_4_ possessed higher photocatalytic activity than N-doped TiO_2_.

**Figure 13:**
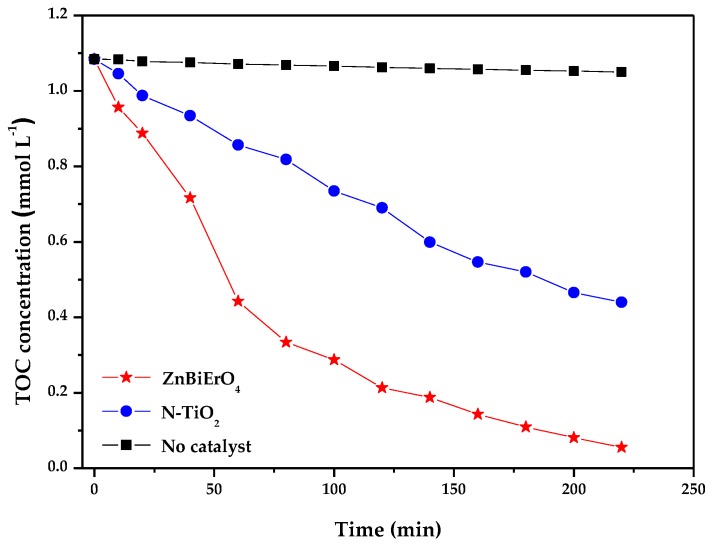
Disappearance of total organic carbon (TOC) during photocatalytic degradation of methyl blue under visible light irradiation in the presence of ZnBiErO_4_ and N-TiO_2_ as well as in the absence of a photocatalyst.

The changes of total organic carbon (TOC) during photocatalytic degradation of MB in the presence of ZnBiErO_4_ and N-TiO_2_ as well as in the absence of a photocatalyst are shown in Figure 13, which was in accordance with the tendency shown in Figure 12. The measurements of TOC represented the change of organic carbon in MB solution which contained ZnBiErO_4_ catalyst, N-doped TiO_2_ catalyst or no catalyst under visible light irradiation. The gradual decrease of TOC represented the gradual disappearance of organic carbon and the reduce rate of TOC was estimated to be 94.87%, 59.42% or 3.09% after visible light irradiation for 220 min in the presence of ZnBiErO_4_ and N-TiO_2_ as well as in the absence of a photocatalyst. Moreover, the reaction stopped when the light was turned off in this experiment, indicating the obvious light response of ZnBiErO_4_ photocatalyst. It was illustrated that MB had been converted to other kinds of byproducts and the organic carbon in the MB had not been decomposed to CO_2_.

**Figure 14:**
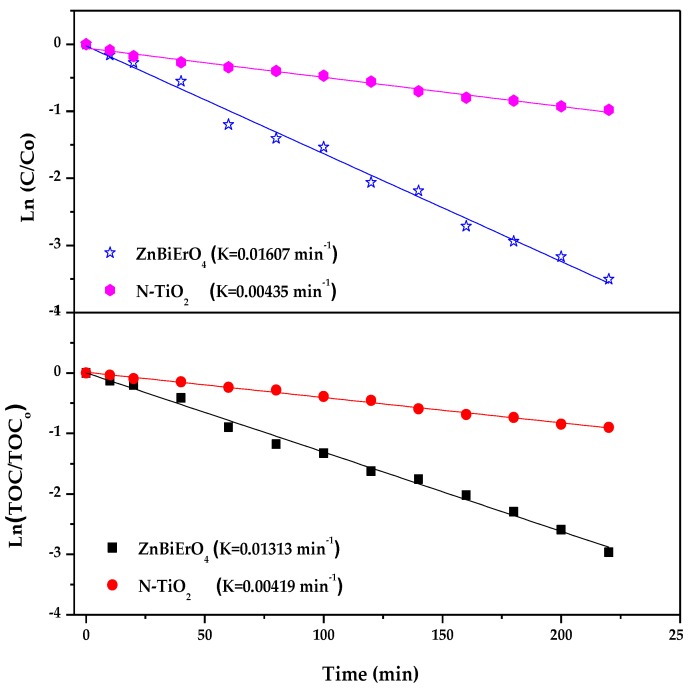
First-order kinetic plots for the photocatalytic degradation of methyl blue with ZnBiErO_4_ or N-TiO_2_ as catalyst under visible light irradiation.

Figure 14 shows the first-order nature of the photocatalytic degradation kinetics with ZnBiErO_4_ or N-doped TiO_2_ as catalyst. A linear correlation between ln (*C/C_o_*) (or ln (*TOC/TOC_o_*)) and the irradiation time could be clearly seen for the photocatalytic degradation of MB by ZnBiErO_4_ or N-doped TiO_2_ under visible light irradiation. According to Figure 14, the first-order rate constant *k_C_* of MB concentration was estimated to be 0.01607 min^−1^ or 0.00435 min^−1^ with ZnBiErO_4 _or N-doped TiO_2 _as catalyst. Based on the result, it was more suitable for ZnBiErO_4 _to degrade MB under visible light irradiation compared with N-doped TiO_2_. Meanwhile, the first-order rate constant *K_TOC_* of TOC was estimated to be 0.01313 min^−1^ or 0.00419 min^−1^ with ZnBiErO_4_ or N-doped TiO_2_ as catalyst. The different value between *k_C_* and *K_TOC_* illustrated that the photodegradation intermediate products of MB probably appeared during the photocatalytic degradation of MB under visible light irradiation. At the same time, ZnBiErO_4_ showed a higher mineralization efficiency for MB degradation compared with N-doped TiO_2_.

**Figure 15:**
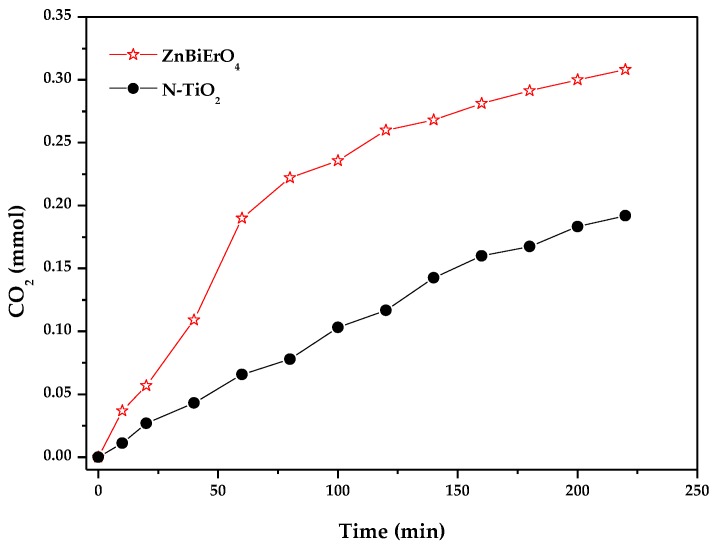
Production of CO_2_ during the photocatalytic degradation of methyl blue with ZnBiErO_4_ or N-TiO_2_ as catalyst under visible light irradiation.

Figure 15 reveals the amount variation of CO_2_ which was yielded during the photodegradation of MB with ZnBiErO_4_ or N-doped TiO_2_ as catalyst under visible light irradiation. The amount of CO_2_ increased gradually with increasing the irradiation time. After visible light irradiation for 220 min, the CO_2_ production of 0.30805 mmol with ZnBiErO_4_ as catalyst was higher than the CO_2_ production of 0.19189 mmol with N-doped TiO_2_ as catalyst. It could be distinctly seen from Figure 13 and Figure 15 that the amount of CO_2_ production was nearly equal to the amount of removed TOC and the quantity of CO_2_ production or the removed TOC was slightly lower than the quantity of reduced MB by using different catalysts. It was demonstrated that MB was mainly mineralized into some inorganic products including CO_2_ and H_2_O.

**Figure 16:**
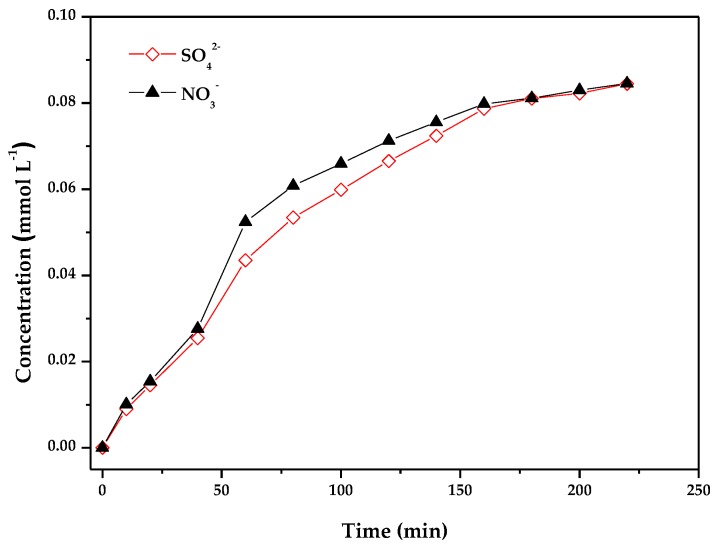
The concentration variation of NO_3_^−^ and SO_4_^2−^ during photocatalytic degradation of methyl with ZnBiErO_4_ as catalyst under visible light irradiation.

The nitrogen and sulfur atoms existed in MB were converted to some inorganic ions such as NO_3_^−^ and SO_4_^2−^ as the end products. Figure 16 shows the concentration variation of NO_3_^−^ ions and SO_4_^2−^ ions when MB was photodegraded by ZnBiErO_4_ under visible light irradiation. As the reaction time went by, it could be clearly seen that the concentration of NO_3_^−^ and SO_4_^2−^ increased gradually during the photocatalytic degradation of MB with ZnBiErO_4_ as catalyst. After visible light irradiation for 220 min, the concentration of SO_4_^2−^ ions was 0.08445 mM with ZnBiErO_4_ as catalyst, indicating that 93.58% of sulfur from MB was converted into sulfate ions with ZnBiErO_4_ as catalyst after visible light irradiation for 220 min. Likewise, the concentration of NO_3_^−^ ions was 0.08456 mM with ZnBiErO_4_ as catalyst during visible light irradiation for 220 min, indicating that 94.46% of nitrogen from MB was converted into nitrate ions with ZnBiErO_4_ as catalyst after visible light irradiation for 220 min. It was worth noting that the amount of SO_4_^2−^ ions released into the solution was lower than the value of stoichiometry. One possible reason might be that part of the sulfur was translated to sulfur-containing volatile compounds such as SO_2_. The second possible reason was that some SO_4_^2−^ ions were absorbed irreversibly on the surface of the photocatalyst, which had been observed by Lachheb et al. [52]. Regardless of the irreversible absorption of sulfate ions on the surface or not, it was important to stress that the evidence for restrained photocatalytic activity was not noticed. In contrast to the sulfate ions, the amount of nitrate ions at the end of the process was found to follow the expected stoichiometric ratio of three. The molecule of MB was converted to small organic species, which were subsequently mineralized into inorganic products such as SO_4_^2−^ ions, NO_3_^−^ ions, CO_2_ and H_2_O ultimately.

**Figure 17:**
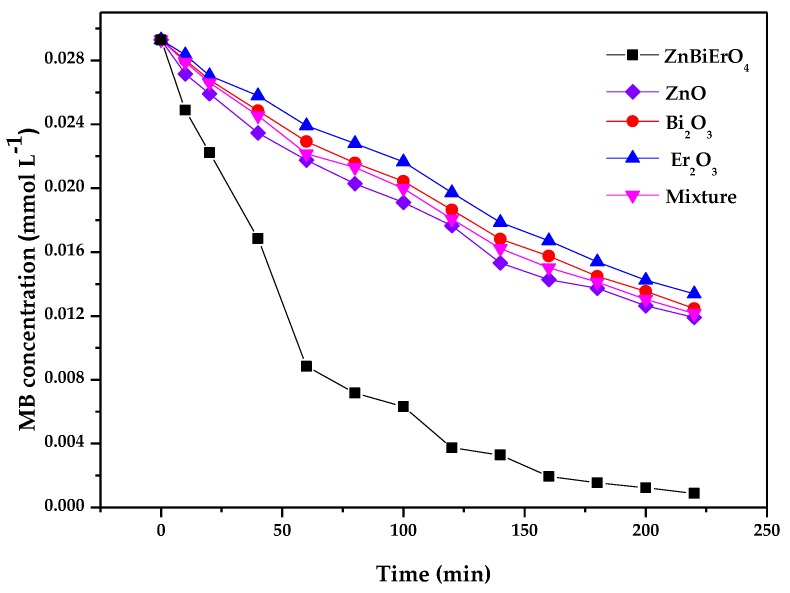
Photocatalytic degradation of methyl blue under visible light irradiation in the presence of ZnBiErO_4_, ZnO, Bi_2_O_3_, Er_2_O_3_ and mixture.

Figure 17 describes the photocatalytic degradation chart of MB under visible light irradiation in the presence of ZnBiErO_4_, ZnO, Bi_2_O_3_, Er_2_O_3_ and mixture which contain ZnO, Bi_2_O_3_ and Er_2_O_3_. After visible light irradiation for 220 min, the removal rate of MB was estimated to be 96.99%, 59.35%, 57.44%, 54.30% or 58.53% with ZnBiErO_4_, ZnO, Bi_2_O_3_, Er_2_O_3_ or mixture as catalyst. It could be observed that the photodegradation rate of MB with ZnBiErO_4_ as catalyst was higher than those with ZnO, Bi_2_O_3_, Er_2_O_3_ or mixture as catalyst, indicating that the photocatalytic degradation activity of ZnBiErO_4_ under visible light was higher than those of ZnO, Bi_2_O_3_, Er_2_O_3_ or mixture. By investigating the photocatalytic degradation results of individual oxides and the synergistic effect of its mixture, we could learn that the photocatalytic activities were improved greatly when the new catalyst was formed, proving the justification of using ZnBiErO_4_ as the photocatalyst.

**Figure 18:**
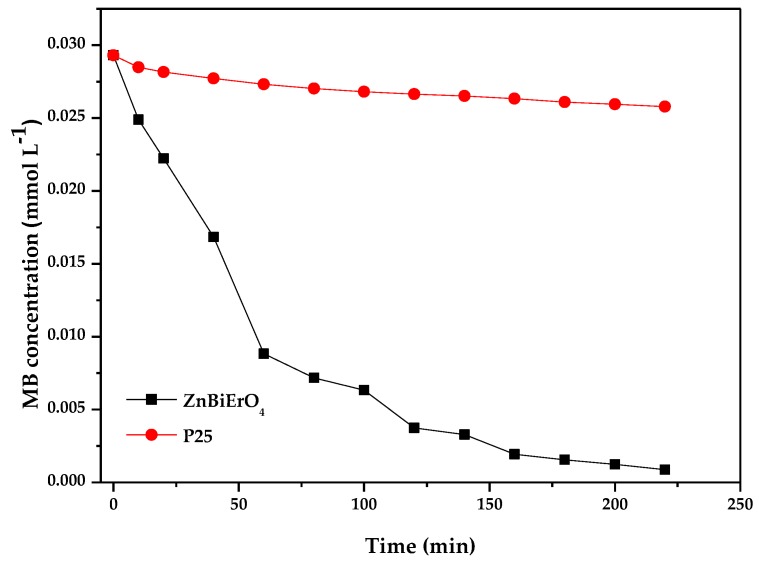
Photocatalytic degradation of methyl blue with ZnBiErO_4_ or P25 as catalyst under visible light irradiation.

**Figure 19:**
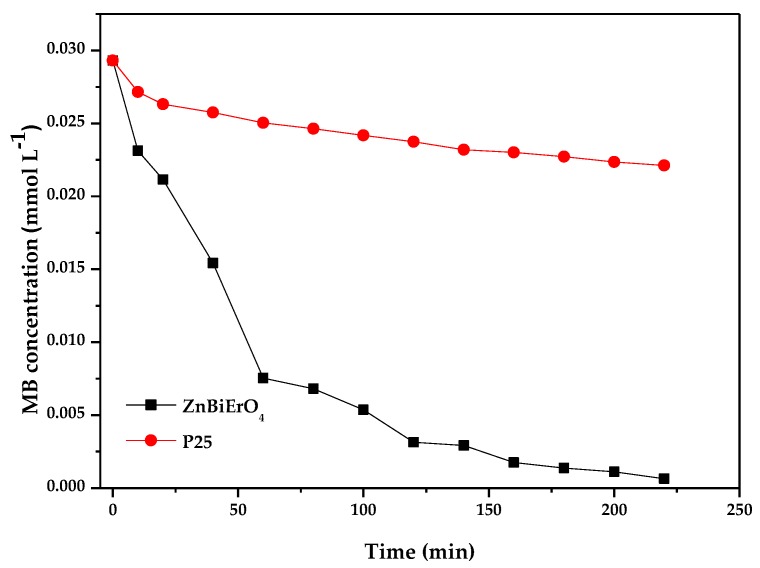
Photocatalytic degradation of methyl blue with ZnBiErO_4_ or P25 as catalyst under sunlight irradiation.

Figure 18 and Figure 19 represent the photocatalytic degradation chart of MB with ZnBiErO_4_ and commercial P25 as catalyst under visible light irradiation or sunlight irradiation. After visible light irradiation for 220 min, the removal rate of MB was estimated to be 96.99% or 12.01% with ZnBiErO_4_ or P25 as catalyst. After sunlight irradiation for 220 min, the removal rate of MB was estimated to be 97.85% or 24.51% with ZnBiErO_4_ or P25 as catalyst. It could be observed that the photodegradation rate of MB with ZnBiErO_4_ as catalyst was higher than those with P25 as catalyst whether under visible light irradiation or under sunlight irradiation, indicating that the photocatalytic degradation activity of ZnBiErO_4_ was higher than those of commercial P25. 

**Figure 20:**
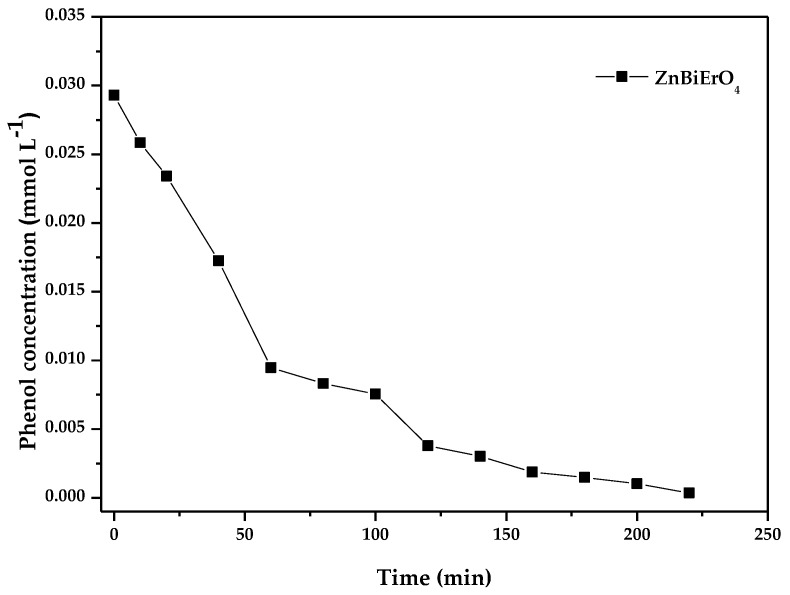
The photocatalytic degradation of phenol with ZnBiErO_4_ as a photocatalyst under visible light irradiation.

Figure 20 shows the photocatalytic degradation of phenol with ZnBiErO_4_ as a photocatalyst under visible light irradiation. According to Figure 20, it was obvious to find that the photocatalytic activity was obtained when colorless phenol was selected as a contaminant model with ZnBiErO_4_ as photocatalyst. The photocatalytic degradation efficiency of phenol was estimated to be 98.80% when ZnBiErO_4_ was used as a photocatalyst under visible light irradiation after 220 min, demonstrating that ZnBiErO_4_ itself had photocatalytic activity and the photosensitive effect was not the main reason in the photodegradation process of MB by using ZnBiErO_4_ as a photocatalyst [53].

### 2.3. Photocatalytic Degradation Mechanism

As well as we have known, the organic pollutants can be degraded by the photogenerated reactive species during the photocatalytic reaction, including electrons (e^−^), holes (h^+^), hydroxyl radicals (•OH) and superoxide radicals (•O_2_^−^). In order to confirm the major reactive species for inducing the degradation of methyl blue with ZnBiErO_4_ as catalyst under visible light irradiation, radical scavenger experiments were performed by adding various scavengers into the system. 

**Figure 21:**
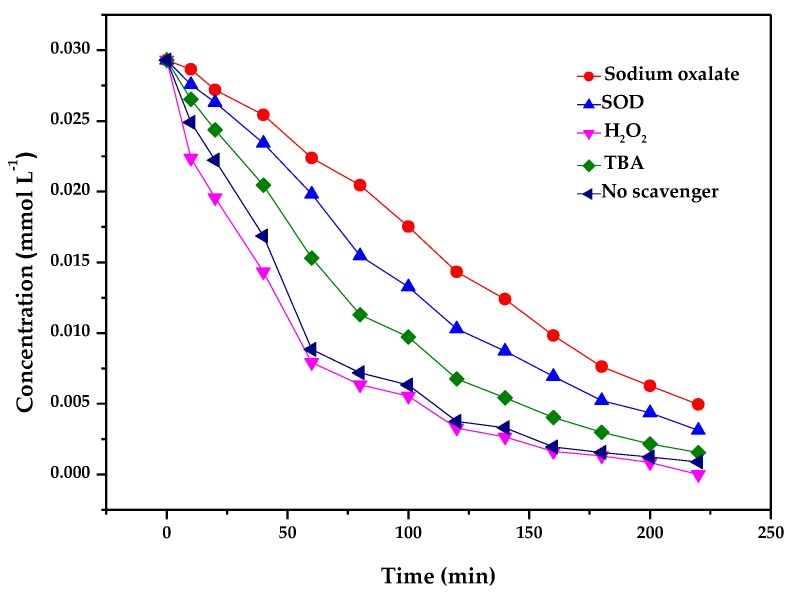
Photodegradation of methyl blue over ZnBiErO_4_ in the presence of various scavengers: sodium oxalate, superoxide dismutase (SOD), H_2_O_2_, tert-butyl alcohol (TBA) and no scavenger.

Superoxide dismutase (SOD) with the concentration of 66.7 mg L^−1^, sodium oxalate with the concentration of 10 mM, H_2_O_2_ with the concentration of 10 mM and tert-butyl alcohol (TBA) with the concentration of 10 mM were added into the system as scavengers to capture •O_2_^−^, h^+^, e^−^ and •OH, respectively. Figure 21 illustrates photodegradation of methyl blue over ZnBiErO_4_ in the presence of various scavengers. As shown in Figure 21, after the addition of H_2_O_2_ as a scavenger for photogenerated electrons (e^−^), the photodegradation efficiency of methyl blue remained almost the same compared with the system of no scavenger, demonstrating the minor role of e^−^ in this process. When tert-butyl alcohol (TBA) was used as the scavenger to capture •OH, the photodegradation activity declined somewhat. However, a significant reduction in the photocatalytic performance was observed in the presence of sodium oxalate and superoxide dismutase (SOD), which captured •O_2_^−^ and h^+^ respectively. The results indicate that the photoreaction process in this system is dominated by h^+^ and •O_2_^−^. In conclusion, h^+^ contribute most to the high activity of the ZnBiErO_4_ during the degradation of methyl blue. Additionally, •O_2_^−^ radicals have a secondary importance during methyl blue degradation. As for •OH, it may participate in the photodegradation process. However, e^−^ exhibit almost no activity during methyl blue degradation in the presence of ZnBiErO_4_.

Based on above results, a possible mechanism of photocatalytic activity enhancement could be proposed as the following steps:

Scheme I:

(R1) $${\mathrm{ZnBiErO}}_{4}\overset{\mathrm{Visible}\;\mathrm{light}}{\to }h^{+}+e^{-}$$

(R2) $$e^{-}+O_{2}\to \cdot O_{2}{}^{-}$$

(R3) $$h^{+}+{\mathrm{OH}}^{-}\to \cdot \mathrm{OH}$$

(R4) $$h^{+}+\cdot \mathrm{OH}+\cdot O_{2}{}^{-}+\mathrm{MB}\to \text{degradation products}$$

Scheme II:

(R5) $${\mathrm{MB}}_{\left(\mathrm{ads}\right)}\overset{\mathrm{Visible}\;\mathrm{light}}{\to }{\mathrm{MB}}_{\left(\mathrm{ads}\right)}^{*}$$

(R6) $${\mathrm{MB}}_{\left(\mathrm{ads}\right)}^{*}+{\mathrm{ZnBiErO}}_{4}\to {\mathrm{ZnBiErO}}_{4}(e)+{\mathrm{MB}}_{\left(\mathrm{ads}\right)}^{+}$$

(R7) $${\mathrm{ZnBiErO}}_{4}(e)+O_{2}\to {\mathrm{ZnBiErO}}_{4}+\cdot O_{2}{}^{-}$$

(R8) $$\cdot O_{2}^{{}^{-}}+\mathrm{MB}\to \mathrm{degradation}\;\mathrm{products}$$

Towards scheme I, the electron-hole pairs were generated on the ZnBiErO_4_ surface upon visible-light excitation (Reaction (R1)), the dissolved oxygen could be activated by the photogenerated electrons to produce superoxide anion radicals (Reaction (R2)), while the holes could react with the absorbed water to form hydroxyl radicals (Reaction (R3)), lastly, the contaminants absorbed on the active sites of ZnBiErO_4_ was oxidized by the active species (holes, superoxide anion radicals and hydroxyl radicals) (Reaction (R4)). The significant enhancement in photoactivity under visible light irradiation could be ascribed to the reduced recombination of photogenerated electrons and photogenerated holes by ZnBiErO_4_ [54].

As for the scheme II, MB adsorbed on the surface ZnBiErO_4_ was excited by visible light irradiation (Reaction (R5)). Subsequently, the electrons were injected from the excited MB to the conduction band of ZnBiErO_4_ (Reaction (R6)), then, the dissolved oxygen could scavenge the electron to produce superoxide anion radicals (Reaction (R7)), finally, the MB contaminants were degraded by the active species of superoxide anion radicals (Reaction (R8)).

**Figure 22:**
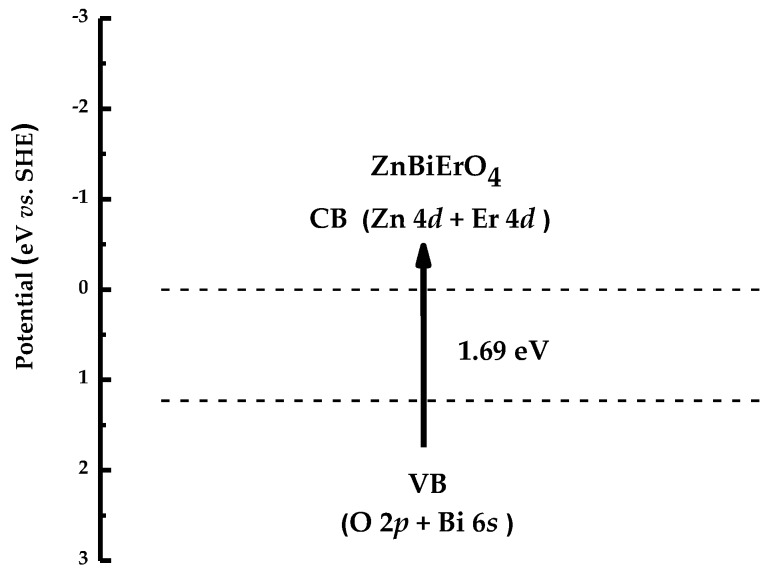
Suggested band structure of ZnBiErO_4_.

Band gap energy indicated the minimum energy needed to photocatalytically degrade MB. Figure 22 shows the band structure of ZnBiErO_4_. The positions and width of the conduction band (CB) and the valence band (VB) were obtained after the calculation of the electronic band structure of ZnBiErO_4_ by the plane-wave-based density functional method. The band structure calculations of ZnBiErO_4_ were carried out with the program of Cambridge serial total energy package (CASTEP) and first principles simulation. According to above method, we deduced that the band gap energy of ZnBiErO_4_ was 1.69 eV, at the same time, the valence band of ZnBiErO_4_ was composed of O 2*p* and Bi 6*s* orbital components and the conduction band of ZnBiErO_4_ was composed of Zn 4*s* and Er 4*d* orbital components. The results indicated that electron-hole pairs could be produced when ZnBiErO_4_ absorbed enough photons which were more than needed photons.

## 3. Experimental

### 3.1. Synthesis of ZnBiErO_4_ and N-Doped TiO_2_

The novel ZnBiErO_4_ photocatalyst was synthesized by a solid-state reaction method. In the first place, ZnO, Bi_2_O_3_ and Er_2_O_3_ with purity of 99.99% (Sinopharm Group Chemical Reagent Co., Ltd., Shanghai, China) were mixed with 2:1.1:1 molar ration to serve as raw materials. All chemicals were dried at 200 °C for 4 h before the preparation of the catalyst. Then the precursors were fully mingled with each other, pressed into small columns and put into an alumina crucible (Shenyang Crucible Co., Ltd., Shenyang, China). Finally, calcination was carried out at 1000 °C for 35 h in an electric furnace (KSL 1700X, Hefei Kejing Materials Technology Co., Ltd., Hefei, China). After sintering and grounding within a quartz mortar, ultrafine ZnBiErO_4_ powder was fabricated. In this study, we prepared about 5 g ZnBiErO_4_ for characterization and experimental and its costs were about four dollars.

Nitrogen-doped titania (N-doped TiO_2_) catalyst was prepared by sol-gel method with tetrabutyl titanate (Shanghai Lingfeng Chemical Reagent Co. Ltd., Shanghai, China) as a precursor and ethanol (Sinopharm Group Chemical Reagent Co., Ltd., Shanghai, China) as solvent. The procedure was as follows: Firstly, 17 mL tetrabutyl titanate and 40 mL absolute ethyl alcohol were combined to serve as to solution A; 40 mL absolute ethyl alcohol, 10 mL glacial acetic acid (Sinopharm Group Chemical Reagent Co., Ltd., Shanghai, China) and 5 mL double distilled water were blended to be solution B; subsequently solution A was added dropwise into solution under vigorous stirring, then a transparent colloidal suspension was formed. Subsequently, aqua ammonia (Shanghai Lingfeng Chemical Reagent Co. Ltd., Shanghai, China), which N/Ti proportion was 8 mol %, was put into the resulting transparent colloidal suspension in the condition of stirring for 1 h. Then the xerogel was generated after being aged for 2 days. The xerogel was grounded into powder which was calcined at 500 °C for 2 h, subsequently above powder was grounded in agate mortar and screened by shaker to obtain N-doped TiO_2_ powders.

### 3.2. Characterization of ZnBiErO_4_

The crystal structures of ZnBiErO_4_ was determined by X-ray diffraction (XRD, D/MAX-RB, Rigaku Corporation, Tokyo, Japan) method, which operated at 40 kV and 40 mA with CuKα radiation (λ = 1.54056 Å). The data were collected at 295 K with a step-scan procedure, whose scan range was 2θ = 5–100°. The step interval was 0.02° and the time per step was 1 s. The chemical composition and the surface structure of the compound was determined by means of scanning electron microscope-X-ray energy dispersion spectrum (SEM-EDS, LEO 1530VP, LEO Corporation, Dresden, Germany). Transmission electron microscopy (TEM, Tecnal F20 S-Twin, FEI Corporation, Hillsboro, OR, USA) was used to observe the surface state and structure of the photocatalysts. The particle sizes of the photocatalysts were measured by Malvern’s mastersize-2000 particle size analyzer (Malvern Instruments Ltd., Malvern, UK). The content of Zn^2+^, Bi^3+^, Er^3+^ and O^2−^ of ZnBiErO_4_ and the valence state of elements were analyzed by X-ray photoelectron spectroscopy (XPS, ESCALABMK-2, VG Scientific Ltd., London, UK). At the same time, X-ray photoelectron spectroscopy also could be utilized to examine the chemical composition within the depth profile of ZnBiErO_4_ using the method of argon ion denudation. The photophysical property of ZnBiErO_4_ was analyzed with an UV-2550 UV-visible spectrophotometer (Shimadzu, UV-2550, Shimadzu Corporation, Kyoto, Japan) in a UV-Vis diffuse reflectance experiment by the dry-pressed disk samples and BaSO_4_ was used as the reference material. 

### 3.3. Photocatalytic Activity Tests

The photocatalytic activity of ZnBiErO_4_ was evaluated by photocatalytic degradation of methyl blue (C_37_H_27_N_3_O_9_S_3_) (Tianjin Bodi Chemical Co., Ltd., Tianjin, China) under visible light. The photoreaction was carried out in a photochemical reaction apparatus (Xujiang Electromechanical Plant, Nanjing, China) with high pressure xenon lamp and Cooling System. The process of our experiments was as follows: 0.3 g photocatalyst powder of N-doped TiO_2_ or ZnBiErO_4_ was added into 300 mL MB aqueous solution in every quartz tube, whose initial concentration was 0.0293 mM and initial pH value was 7.0. In order to ensure the establishment of an adsorption/desorption equilibrium among photocatalysts, the MB dye and atmospheric oxygen, every solution was magnetically stirred in the dark for 45 min. In this paper, a 500 W xenon lamp was used as a light source with a 400 nm cutoff filter to provide visible light irradiation. During visible light illumination, the MB dye pollution was stirred by a magnetic stirrer with 500 rpm and the photocatalyst powder was kept suspended in the solution. One of the quartz tubes was taken out from the photochemical reaction apparatus at various time intervals. Subsequently the suspension was filtered through 0.22 µm membrane filters and the filtrate was analyzed by a Shimadzu UV-2450 UV-Visible spectrometer with the detecting wavelength at 591 nm. Above experiments were performed under oxygen-saturation conditions ([O_2_] sat = 1.02 × 10^−3^ M), pH adjustment was not carried out and the initial pH value was 7.0 and a near constant reaction temperature (20 °C) was maintained by recycling water.

The identification of MB and the intermediate products of MB were measured by liquid chromatograph-mass spectrometer (LC-MS, Thermo Quest LCQ Duo, Thermo Fisher Scientific Corporation, Silicon Valley, CA, USA. Beta Basic-C18 HPLC column: 150 × 2.1 mm, ID of 5 μm, Thermo Fisher Scientific Corporation, Silicon Valley, CA, USA). There were post-photocatalysis solution (20 μL) in the LC-MS system, which were injected automatically. The fluent was composed of 60% methanol and 40% water and the flow rate was 0.2 mL·min^−1^. MS conditions consisted of an electrospray ionization interface, a capillary temperature of 27 °C with a voltage of 19.00 V, a spray voltage of 5000 V and a constant sheath gas flow rate. The negative ion scan mode and the *m*/*z* range swept from 50 to 600 in the spectrum were acquired. The concentration of total organic carbon (TOC) was determined with a TOC analyzer (TOC-5000, Shimadzu Corporation, Kyoto, Japan). Evolution of CO_2_ was measured with an intersmat^TM^ IGC120-MB gas chromatograph (Thermo Separation Products Corporation, Brussels, Belgium). The device equipped with a porapack Q column (3 m in length and with an inner diameter of 0.2 in), which was connected to a catharometer detector. The obtained inorganic products from MB degradation were analyzed by ion chromatograph (DX-300, Dionex Corporation, Sunnyvale, CA, USA). The photonic efficiency was calculated according to the following equation [55,56]:

*ξ* = *R*/*I*_0_ (2)

where *ξ* is the photonic efficiency (%), *R* is the rate of MB degradation (Mol L^−1^ s^−1^) and *I*_0_ is the incident photon flux (Einstein·L^−1^ s^−1^). The incident photon flux *I*_0_ measured by a radiometer (Model FZ-A, Photoelectric Instrument Factory Beijing Normal University, Beijing, China) was determined to be 4.76 × 10^−6^ Einstein·L^−1^·s^−1^ under visible light irradiation.

## 4. Conclusions

In our study, ZnBiErO_4_ was prepared by a solid-state reaction method for the first time. The structural properties of ZnBiErO_4_ was characterized by some material characterization methods and the photocatalytic properties of ZnBiErO_4_ was also verified in comparison with N-doped TiO_2_. XRD results indicated a good agreement between the observed and calculated intensities in a tetragonal crystal structure with space group I41/A. The latticeparameters for ZnBiErO_4_ were proved to be a = b = 10.255738 Å and c = 9.938888 Å. XPS results confirmed that the synthesized ZnBiErO_4_ was highly pure under our preparation conditions. The band gap of ZnBiErO_4_ was estimated to be about 1.69 eV, indicating that ZnBiErO_4_ could absorb visible light (*λ* > 400 nm). Photocatalytic decomposition of aqueous MB was realized under visible light irradiation in the presence of ZnBiErO_4_ and N-doped TiO_2_. The results could obviously tell that the photodegradation rate of MB and the photonic efficiency with ZnBiErO_4_ as catalyst was higher than those with N-doped TiO_2_, which illustrated that ZnBiErO_4_ exhibited higher photocatalytic activities for MB degradation under visible light irradiation compared with N-doped TiO_2_. The photocatalytic degradation of MB with ZnBiErO_4_ or N-doped TiO_2_ as catalyst followed the first-order reaction kinetics. The apparent first-order rate constant of ZnBiErO_4_ or N-doped TiO_2_ was 0.01607 min^−1^ or 0.00435 min^−1^. The reduction of total organic carbon, formation of inorganic products such as SO_4_^2−^ and NO_3_^−^ and the evolution of CO_2_ revealed the continuous mineralization of MB during the photocatalytic process. In addition, the possible photocatalytic degradation pathway of MB was deduced under visible light irradiation. Otherwise, the costs (about 0.8 dollars per gram) of ZnBiErO_4_ photocatalyst are low and the prepared materials are available, proving the availability and feasibility for a large scale application. In conclusion, the results indicated that ZnBiErO_4_/visible light photocatalysis system might be regarded as a practical method for the treatment of dye wastewater. As this system did not need high pressure of oxygen, heating or any chemical reagents, it has a promising application in purifying and reusing colored aqueous effluents.

## Figures and Tables

**Table 1:** Atomic coordinates and structural parameters of ZnBiErO_4_ prepared by the solid reaction method.

Atom	x	y	z	Occupation Factor
Zn	0	0	0.5	1
Bi	0	0	0	1
Er	0	0	0	1
O	0.76731	0.14013	0.08188	1

**Table 2:** Binding energies (BE) for key elements from ZnBiErO_4_.

Compound	Zn2p_1/2_ BE (eV)	Zn2p_3/2_ BE (eV)	Bi4f_7/2_ BE (eV)	Bi4f_5/2_ BE (eV)	Er4d BE (eV)	O1s BE (eV)
ZnBiErO_4_	1043.6	1020.3	157.7	163.0	162.9	529.8
